# Transcriptome-and Metabolome-Based Mechanisms of High-Temperature Adaptation in Triploid Rainbow Trout (*Oncorhynchus mykiss*)

**DOI:** 10.3390/biology15141194

**Published:** 2026-07-20

**Authors:** Shuchen Huang, Changzhong Li, Ying Yang, Tianxiu Liang, Xin Xie, Jingjing Zhang, Zhaonan Li, Jin Li, Yanxia Chen

**Affiliations:** 1 College of Eco-Environmental Engineering, Qinghai University; Xining 810016, China; 19544523629@163.com (S.H.); lichangzhong@qhu.edu.cn (C.L.); y282938127@163.com (Y.Y.); 18093980289@163.com (T.L.); 17538243413@163.com (X.X.); 18293624272@163.com (J.Z.); lizhaonan123456@163.com (Z.L.); 2 Key Laboratory of Plateau Cold-Water Fish Culture and Eco-Environmental Conservation (Co-Construction by Ministry and Province), Ministry of Agriculture and Rural, Xining 810016, China; 3 Qinghai Key Laboratory of Breeding and Protection of Naked Carp, Qinghai Lake Naked Carp Rescue Center, Xining 810016, China; lijinyxyu@163.com

**Keywords:** high-temperature adaptation, body weight classes, transcriptomics

## Abstract

High-temperature stress poses a critical challenge to cold-water aquaculture; however, the size-associated molecular mechanisms underlying thermal adaptation in triploid rainbow trout (*Oncorhynchus mykiss*) remain poorly understood. Here, we integrated transcriptomic and metabolomic profiling of liver tissues, which plays central role in energy metabolism and stress integration, from three body-weight classes—small (0.8 kg ± 0.18 kg), medium (1.5 kg ± 0.22 kg), and large (2.5 kg ± 0.31 kg)—sampled under peak summer heat stress (20.5 °C). Transcriptomic analysis identified 974, 570, and 862 group-specific differentially expressed genes, respectively, revealing a non-linear, size-associated transcriptional pattern. Genes assigned to the ribosome pathway were significantly enriched in comparisons involving MLA and SLA relative to LLA, but not between SLA and MLA, whereas carbon metabolism and amino acid biosynthesis were enriched exclusively in smaller fish, indicating a higher catabolic burden under heat stress. Metabolomic profiling identified 1123 metabolites, with lipids accounting for 45.06%, and showed size-specific enrichments in biosynthesis of unsaturated fatty acids, glycerophospholipid metabolism, arachidonic acid metabolism, and pathways related to necroptosis. Integrative analysis revealed that in smaller fish, *pla2g1b* and *gpx4a* coordinately regulate the accumulation of prostaglandin H2 and 16(R)-HETE, forming a regulatory network with ferroptosis-related genes *acsl4a* and *hmox1a*; concurrently, *chka*, *lpin1*, and phospholipase A2 members drive extensive membrane phospholipid remodeling. The observed negative correlation between *hsd17b3* expression and 7α-hydroxytestosterone levels suggests size-associated steroid-mediated energy repartitioning. Collectively, smaller fish undergo extensive transcriptional and metabolic reprogramming with heightened activation of cell death pathways, whereas larger fish maintain greater thermal buffering capacity. These findings provide molecular targets for size-stratified thermal management and selective breeding in rainbow trout aquaculture.

## Simple Summary

Rainbow trout is one of the world’s most important farmed fish, but as a cold-water species it is highly sensitive to rising water temperatures caused by climate change. Farmers often raise triploid rainbow trout—fish with an extra set of chromosomes that cannot reproduce naturally—because they grow faster and produce higher-quality flesh. However, during hot summer months, these fish commonly cease feeding, exhibit stunted growth, and die in large numbers, causing serious economic losses. A key but poorly understood question is whether fish of different body sizes respond differently to heat stress at the molecular level. In this study, we collected liver tissue from small (0.8 kg ± 0.18 kg), medium (1.5 kg ± 0.22 kg), and large (2.5 kg ± 0.31 kg) triploid rainbow trout during peak summer heat at a major farming site in northwest China, employing advanced molecular profiling techniques to simultaneously quantify thousands of genes and metabolites. We found that smaller fish underwent the most dramatic molecular changes, activating emergency energy production, extensively remodeling cell membranes, and initiating stress-related cell death programs. Medium-sized fish primarily modulated the fatty acid composition of their membranes to adapt to heat, while larger fish showed the most stable molecular responses. These size-specific differences identify potential molecular targets that could be used to develop better farming management strategies and breed more heat-tolerant triploid rainbow trout, supporting more sustainable cold-water aquaculture.

## 1. Introduction

Rainbow trout (*Oncorhynchus mykiss*), a salmonid species native to the Pacific coast of North America, stands as one of the world’s most commercially significant food fish—globally, aqua-culture production surpassed 195 million tons in 2024, with farmed finfish accounting for over 103 million tons, and within this sector rainbow trout consistently ranks among the top farmed species, with major producers including Chile, Norway, Turkey, Iran, and Russia [1,2,3]. Rainbow trout farming spans more than 70 countries with an annual production exceeding 800,000 metric tons, making it the second most cultured salmonid species after Atlantic salmon (Salmo salar). This species is prized not only for its adaptability to diverse rearing systems (ponds, raceways, cages, and recirculating systems) but also for its high consumer acceptance and nutritional value [4].

Triploid rainbow trout (*Oncorhynchus mykiss*) are artificially induced polyploids that carry three complete sets of chromosomes, resulting in genomic triploidy. This chromosomal configuration leads to reproductive sterility and an inability to reproduce naturally [5,6]. The sterility characteristic offers notable benefits in aquaculture settings. As energy typically allocated to gonadal development and reproduction is reallocated to somatic growth, triploid rainbow trout exhibit enhanced physiological robustness, accelerated growth rates, and superior filet quality—the latter reflected in higher crude fat content and improved color characteristics compared with diploids [7]. Bio-economic modeling has further demonstrated that rearing triploids can be more profitable than diploids, primarily through improved survival rates and increased product value [8]. Additionally, their reproductive sterility reduces the environmental risks posed by farmed escapees. In Chilean Patagonia, for instance, escaped diploid farmed rainbow trout have hybridized with naturalized wild populations at frequencies ranging from 7% to 69%, substantially increasing population admixture [9]. In the Rocky Mountain drainages of North America, introgressive hybridization between non-native rainbow trout and native westslope cutthroat trout (*Oncorhynchus clarkii lewisi*) has been extensively documented, with even modest levels of admixture reducing the reproductive fitness of hybridized individuals by approximately 50% [10]. In the Lake Superior basin, hatchery-origin rainbow trout have introgressed into naturalized steelhead populations, with average kamloops ancestry reaching 8% in juvenile fish from affected streams [11]. Similarly, in Mexico, cultured rainbow trout have introgressed with native trout species, threatening the genetic integrity of endemic lineages [12]. These cases underscore the ecological value of triploidy as a containment strategy: because triploids cannot reproduce, they pose no risk of genetic introgression even if they escape into the wild [13]. Consequently, triploid rainbow trout demonstrate enhanced economic value in commercial aquaculture [14,15,16].

As a cold-water species, *O. mykiss* are highly sensitive to water temperature, typically surviving at temperatures ranging from 0 to 26 °C and exhibiting optimal growth between 12 and 18 °C [17]. When temperatures exceed this optimal range, physiological stress responses are triggered, leading to reduced growth performance and increased mortality risk [18,19,20]. At the molecular and metabolic levels, elevated temperatures induce extensive alterations in gene expression patterns and metabolic pathways in aquatic animals. Heat stress triggers the upregulation of heat shock proteins (HSPs), oxidative stress-related genes, and immune response genes, while simultaneously disrupting energy metabolism, protein synthesis, and cellular homeostasis [21,22,23]. Among HSPs, hsp70 and hsp90 families have been most extensively characterized, with multiple isoforms showing tissue- and time-specific expression patterns under heat exposure [24]. Genome-wide analyses have further identified additional HSP family members—such as hsp40—that exhibit tissue-specific upregulation (e.g., liver and head kidney) following heat challenge [25]. Among immune-related genes, the pro-inflammatory cytokine interleukin-1β (il-1β) is markedly upregulated under thermal stress, with sensitive individuals showing higher expression than tolerant ones [26]. The chemokine IL-8 and hypoxia-inducible factor hif-1α are also significantly induced, reflecting an inflammatory response and oxygen-sensing pathway activation that may compromise disease resistance [27]. These immune disruptions are particularly relevant in densely stocked cage and pond systems, where elevated pathogen exposure makes immune competence a critical determinant of production outcomes [28]. These molecular responses reflect the organism’s attempt to cope with thermal challenges but often incur significant energetic costs, further compromising growth and survival [29].

Previous studies in diploid rainbow trout and other teleost species have established that heat stress induces characteristic metabolic reprogramming at the hepatic level, which has informed our experimental design. In diploid rainbow trout subjected to acute thermal challenge, metabolomic profiling of the liver has revealed coordinated changes in energy metabolism pathways—including tricarboxylic acid cycle intermediates, amino acid catabolism products, and purine metabolites—that collectively reflect a systemic shift toward enhanced energy mobilization at the expense of growth-related anabolism [30]. Similar patterns have been documented in other thermally stressed fish: in turbot (*Scophthalmus maximus*), heat stress activates lipid catabolism through the PPARβ signaling pathway, upregulating fatty acid β-oxidation genes such as *acox* and *cpt-1* to meet elevated energy demands [31]. In rainbow trout (*Oncorhynchus mykiss*), chronic high-temperature exposure triggers hepatic lipid metabolism disorder and ferroptosis via oxidative-stress-mediated pathways [30]. These studies collectively demonstrate that lipid remodeling, amino acid catabolism, and oxidative stress responses represent conserved metabolic hallmarks of thermal stress across diverse fish taxa. However, whether these metabolic signatures are modulated by body size within a single population, and whether triploidy introduces ploidy-specific features that alter these conserved responses, have not been systematically investigated.

Amid global climate change, heat stress has become a critical challenge for cold-water aquaculture, with seasonal temperature extremes severely constraining rainbow trout production efficiency [32]. The Longyangxia Reservoir, located on the upper Yellow River, serves as a major production area for triploid rainbow trout and is characterized by significant seasonal temperature variation [33,34]. The reservoir exhibits pronounced seasonal thermal dynamics owing to its high-altitude setting and large storage capacity. Instrumental monitoring and three-dimensional modeling have shown that during summer (June to September), surface water temperatures reach 16–21 °C, while during winter (December to March), the water column becomes nearly isothermal at approximately 5–6 °C, a result of the reservoir’s multi-year regulating function that releases warm water in winter and cold water in summer [35,36,37]. Studies have shown that during high-temperature periods in August, farmed triploid rainbow trout in this region commonly exhibit reduced feed intake, impaired growth, and increased mortality [35,36,37],which substantially undermines economic viability. Although thermal tolerance is known to vary with fish size, systematic studies on size-associated physiological responses to summer heat stress in triploid rainbow trout remain scarce.

The liver is the principal organ governing energy metabolism, biotransformation, and systemic stress responses in teleost fish [38]. In rainbow trout, hepatic tissue responds rapidly to elevated temperature, with heat stress altering the expression of genes involved in protein processing, energy homeostasis, and lipid metabolism [39]. The liver also serves as the primary site for lipid synthesis and remodeling—processes central to thermal acclimation [40]. Compared with skeletal muscle, which largely reflects structural properties, the liver offers a more direct readout of systemic metabolism and stress-coping capacity. Moreover, the hepatosomatic index (HSI) scales with body size and nutritional status, making the liver a reliable tissue for capturing size-related differences in metabolic state [41] We therefore selected liver tissue for multi-omics profiling to establish a basis for comparing heat stress responses among differently sized triploid rainbow trout.

This study utilized integrated transcriptomic and metabolomic approaches to characterize hepatic gene expression and metabolite profiles in size-stratified triploid rainbow trout (*Oncorhynchus mykiss*) subjected to heat stress induced in August. By combining transcriptome and metabolome data, this study establishes a comparative framework to elucidate size-associated molecular and metabolic responses to heat stress and uncover potential thermal adaptation strategies. These findings provide a molecular and metabolic foundation for developing size-specific thermal tolerance markers and precision management practices, thereby enhancing resilience to seasonal heat stress and supporting the sustainable development of the triploid rainbow trout aquaculture industry.

## 2. Materials and Methods

### 2.1. Experimental Animals and Sample Collection

In this study, all triploid rainbow trout originated from a single full-sibling family produced at Qinghai Minze Longyangxia Ecological Aquatic Products Co., Ltd. in Longyangxia Town, Gonghe County, Hainan Tibetan Autonomous Prefecture, Qinghai Province, China. and reared in circular commercial net cages (100 m in circumference). Throughout the rearing period, the fish were continuously fed a commercial diet formulated with 41% crude protein and 23% crude lipid, and all husbandry procedures adhered to standard commercial production protocols. At sampling (August 2023), the ambient water temperature was 20.5 °C, and the dissolved oxygen concentration was approximately 5.7 mg/L. Fish were classified into three size classes according to body weight: small (0.8 ± 0.18 kg, SLA), medium (1.5 ± 0.22 kg, MLA), and large (2.5 ± 0.31 kg, LLA). Six individuals from each size group were collected during the same harvesting event using standard commercial nets. Prior to sampling, all fish were maintained under identical rearing conditions and fed an identical commercial diet.

Prior to tissue collection, fish were fasted for 24 h to standardize their metabolic state. All individuals were then anesthetized with eugenol (1:10,000 *v*/*v*; Shanghai Reagent Corporation, Shanghai, China). Liver tissues were dissected immediately, rapidly frozen in liquid nitrogen, and subsequently stored at −80 °C for further analysis.

### 2.2. Transcriptome Analysis

Total RNA was isolated from triploid rainbow trout liver samples using TRIzol reagent (Thermo Fisher Scientific Inc., Waltham, MA, USA). The concentration, purity, and integrity of the RNA samples were assessed using a spectrophotometer (NanoDrop ND-2000, Thermo Fisher Scientific Inc., Waltham, MA, USA) and an Agilent 2100 Bioanalyzer (Santa Clara Agilent Technologies Inc., CA, USA). For subsequent library construction, RNA samples were required to meet the following quality criteria: an OD260/OD280 ratio between 1.8 and 2.0, and an RNA Integrity Number (RIN) greater than 7.0. RNA-seq libraries were constructed using the NEBNext Ultra RNA Library Prep Kit (New England Biolabs Inc., Ipswich, MA, USA). Library quality was assessed on an Agilent Bioanalyzer 2100 system. Qualified libraries were then sequenced on an Illumina NovaSeq 6000 platform (Illumina Inc., San Diego, CA, USA) to generate raw reads.

The obtained raw reads (FASTQ format) were quality-controlled using fastp (v0.23.0) to remove reads containing adapters, poly-N sequences, and low-quality reads, generating clean reads. Q20, Q30, and GC content of the clean data were calculated simultaneously. The reference genome and gene model annotation files for triploid rainbow trout (GCF_013265735.2) were downloaded from the genome database. Clean reads were then aligned to the reference genome using HISAT2 (v2.0.5). Gene expression levels were quantified using fragments per kilobase per million mapped reads (FPKM). Differentially expressed genes (DEGs) in liver tissue were identified from raw read count data using the DESeq2 package [42], with genes meeting the thresholds of *p* < 0.05 and |Fold Change| ≥ 2 classified as DEGs. Functional profiling of these DEGs was conducted via Gene Ontology (GO) and Kyoto Encyclopedia of Genes and Genomes (KEGG) pathway enrichment analyses implemented in R.

### 2.3. Quantitative Real-Time PCR (RT-qPCR) Validation

To validate the reliability of the transcriptomic data, selected differentially expressed genes (DEGs) were further examined by RT-qPCR. Total RNA was extracted from the same liver samples used for RNA-seq using TRIzol reagent (Thermo Fisher Scientific, USA) following the manufacturer’s protocol. RNA concentration and purity were assessed using a NanoDrop ND-2000 spectrophotometer, and RNA integrity was verified on an Agilent 2100 Bioanalyzer; only samples with an OD260/OD280 ratio between 1.8 and 2.0 and an RNA Integrity Number (RIN) ≥ 7.0 were used for subsequent analysis. First-strand cDNA was synthesized from 1 μg of total RNA using the PrimeScript^TM^ RT Reagent Kit with gDNA Eraser (Takara Bio Inc., Kusatsu, Shiga, Japan) according to the manufacturer’s instructions. The cDNA products were diluted to a working concentration of 100 ng/μL and stored at −20 °C until use.

Primers for the selected genes were designed using Primer Premier 5.0 (Table 1) and beta-actin (*β-actin*) genes were selected as reference genes based on previously established stability in triploid rainbow trout tissues. RT-qPCR was performed on a QuantStudio 6 Flex Real-Time PCR System (Applied Biosystems, Waltham, MA, USA) using TB Green^®^ Premix Ex Taq^TM^ II (Takara Bio Inc., Kusatsu, Shiga, Japan) in a 20 μL reaction volume containing 10 μL of 2× SYBR Green mix, 0.5 μL each of forward and reverse primers (10 μM), 2 μL of diluted cDNA, and 7 μL of RNase-free water. The thermal cycling conditions were as follows: initial denaturation at 95 °C for 30 s, followed by 40 cycles of 95 °C for 5 s and 60 °C for 30 s. A dissociation curve analysis was conducted after each run to confirm the specificity of amplification. Each sample was run in triplicate, and the relative RNA levels of target genes were calculated using the 2^−ΔΔCt^ method [43]. Data are presented as mean ± SD (*n* = 3 per group), and pairwise comparisons were performed to detect differences between individual size groups (SLA vs. LLA and MLA vs. LLA) with one-way ANOVA testing, as the expression patterns were not uniformly monotonic across all genes examined.

### 2.4. LC-MS/MS Analyses

Endogenous metabolites from liver samples were analyzed using untargeted liquid chromatography–tandem mass spectrometry (LC-MS/MS), and the resulting data were handled following the methodologies outlined in earlier studies [44,45].

To extract metabolites, a 100 mg tissue sample was ground using liquid nitrogen and transferred to an EP tube, where it was mixed with 500 μL of an 80% methanol solution. The mixture was vortexed and shaken and then placed in an ice bath for 5 min before being centrifuged at 15,000× *g* for 20 min at 4 °C. The resulting supernatant was diluted with mass spectrometry-grade water, which was obtained from Merck (Darmstadt, Germany, catalog No. 1.15333.2500), to achieve a 53% methanol concentration. Following this, a centrifugation step at 5000× *g* for 20 min at 4 °C was conducted, and the supernatant was collected for analysis via LC-MS.

Chromatographic Setup. The column utilized was a Hypesil Gold C18. The mobile phases included Phase A, which consisted of 0.1% formic acid, and Phase B, which was methanol. The gradient elution was executed as follows: at 0 min, the ratio of A to B was 98:2 (*V*/*V*); this was maintained at 1.5 min, shifted to 15:85 (*V*/*V*) at 3 min, reached 0:100 (*V*/*V*) by 10 min, reverted to 98:2 (*V*/*V*) at 10.1 min, and remained at 98:2 (*V*/*V*) until 12 min. The flow rate was set at 0.2 mL/min, the column temperature was maintained at 40 °C, and the injection volume was 4 μL.

Mass Spectrometry Parameters. The scanning range utilized was *m*/*z* 100–1500. The settings for the ESI source included: spray voltage at 3.5 kV; sheath gas flow rate at 35 psi; auxiliary gas flow rate at 10 L/min; capillary temperature at 320 °C; S-lens RF level set to 60; auxiliary gas heater temperature at 350 °C; and polarity set to both positive and negative. Mass spectrometry analysis was conducted using a data-dependent acquisition (DDA) strategy, a widely employed approach in untargeted metabolomics for comprehensive metabolite profiling.

The analysis and visualization of metabolomics data were conducted utilizing Excel 2019, GraphPad Prism 10, R (version 4.3.1), and SPSS (version 26.0). Mass spectrometry data underwent processing with CD3.1 software, where parameters such as retention time and mass–charge ratio for each metabolite were evaluated. Identification and quantitative assessment of metabolites from the samples were carried out using the mzCloud (RRID:SCR_014669, https://www.mzcloud.org/) [46], mzVault (Thermo Fisher Scientific, mzVault Spectral Libraries, https://www.thermofisher.com/) [47], and Masslist databases (MassList Database, http://www.maldi-msi.org/mass) [48]. Principal component analysis (PCA) and orthogonal partial least squares–discriminant analysis (OPLS-DA) were executed with the metaX software (version 2.0.0) to determine the variable importance in projection (VIP) for each metabolite. OPLS-DA models were validated by 200-fold random permutation testing. For each iteration, the group labels were scrambled, and the model was refitted using the same parameters (including the same number of predictive and orthogonal components determined by cross-validation). The resulting R^2^Y and Q^2^Y values from permuted models were compared with those from the original unpermuted model. This approach has been widely adopted to assess model overfitting and to confirm that the observed group separation is not a spurious result of the data structure. The statistical significance (*p* value) and fold change (FC value) for each metabolite were derived from t-tests. Differentially abundant metabolites (DAMs) were identified based on the criteria of VIP > 1, *p* < 0.05, and a fold change (FC) of either ≥2 or ≤0.5. Finally, functional annotation of the identified DAMs was conducted using the KEGG database.

### 2.5. Integrated Analysis of Multi-Omics Data

To explore the functional relationships between transcriptomic and metabolomic alterations, we performed integrated correlation analyses focusing on pathways that were significantly enriched in both DEGs and DAMs within each pairwise comparison (SLA vs. LLA, MLA vs. LLA, and SLA vs. MLA). For each comparison, we first identified KEGG pathways that showed significant enrichment in both omics datasets (*p* < 0.05). Within each co-enriched pathway, we extracted the DEGs and DAMs annotated to that pathway and calculated Pearson correlation coefficients between their expression levels (FPKM values for genes) and relative abundances (for metabolites). The resulting gene–metabolite correlation matrices were visualized as clustered heatmaps using the ComplexHeatmap package (version 2.18.0) in R (version 4.3.1).

## 3. Results

### 3.1. Feature Analysis of Transcriptomic Data

Around 149.57 Gb of premium data was collected from the liver tissues of triploid rainbow trout, with each individual sample producing at least 5.62 Gb. In every sample, the Q30 base percentage surpassed 94.01% (Table 2).

**Figure 1:**
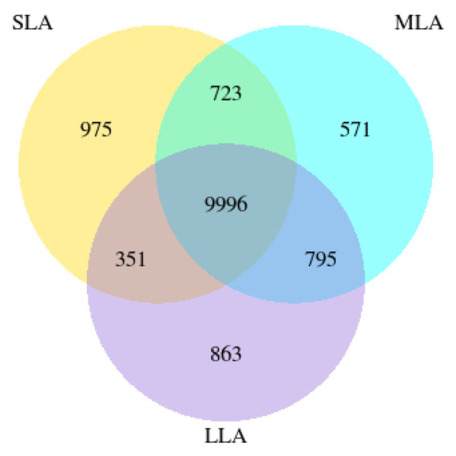
Venn diagram of detected genes in the liver tissues triploid rainbow trout. Each circle represents one size group. Numbers in non-overlapping regions indicate genes expressed exclusively in that group, whereas numbers in overlapping regions indicate genes co-expressed between or among the corresponding groups. A complete list of gene IDs and annotations for all expressed genes is provided in Supplementary Table S1.

In this study, there were 12,045, 12,085 and 12,005 unigenes detected in the SLA, MLA and LLA groups, respectively. Among these, 975, 571, and 863 genes were unique to SLA, MLA, and LLA, respectively. A total of 9996 genes were commonly expressed in all three groups. Furthermore, pairwise overlaps revealed 723 genes co-expressed in both SLA and MLA, 795 co-expressed in both MLA and LLA, and 351 co-expressed in both SLA and LLA groups (Figure 1).

### 3.2. Analysis of Differentially Expressed Genes

**Figure 2:**
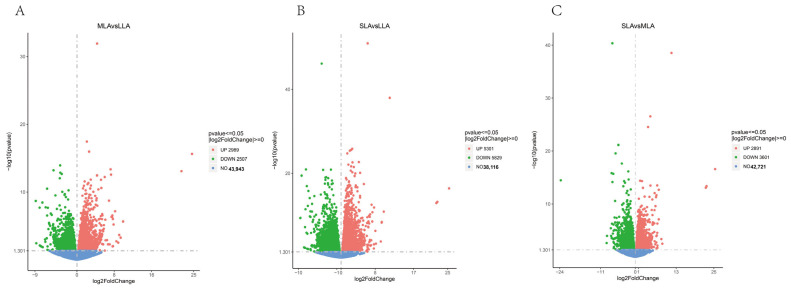
Volcano plots of DEGs between different size groups. (**A**) MLA vs. LLA; (**B**) SLA vs. LLA; (**C**) SLA vs. MLA. Red points represent up-regulated genes, green points represent down-regulated genes, and blue points indicate genes with no significant difference. The detailed lists of differentially expressed genes are provided in Supplementary Table S2.

In the analysis contrasting MLA and LLA, a total of 5496 differentially expressed genes (DEGs) were found, with 2989 showing increased expression and 2507 showing decreased expression (Figure 2A). When examining the SLA and LLA groups, 11,130 DEGs were identified, comprising 5301 that were up-regulated and 5829 that were down-regulated (Figure 2B). The comparison between SLA and MLA revealed 6492 DEGs, which included 2891 up-regulated and 3601 down-regulated genes (Figure 2C). Both MLA and SLA displayed a greater number of down-regulated genes in response to heat stress during the summer when compared to the LLA group, highlighting a significant transcriptional reaction to increased temperatures.

### 3.3. GO Enrichment of Differentially Expressed Genes

**Figure 3:**
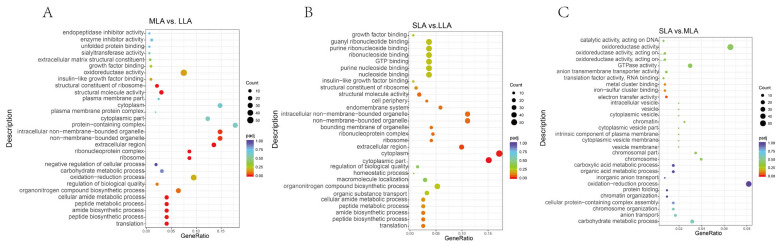
Bubble charts of Gene Ontology (GO) enrichment analysis for DEGs. (**A**): GO enrichment bubble chart for DEGs in MLA vs. LLA; (**B**): GO enrichment bubble chart for DEGs in SLA vs. LLA; (**C**): GO enrichment bubble chart for DEGs in SLA vs. MLA. The size of the bubbles represents the number of genes, and the color indicates the enrichment significance (−log10 (*p*-value)). The detailed lists of GO enrichment of differentially expressed genes are provided in Supplementary Table S3.

In the analysis comparing MLA and LLA, differentially expressed genes (DEGs) showed notable enrichment in biological processes such as “translation”, “peptide biosynthesis”, and “peptide metabolism”, along with cellular components like “ribosomes”, “ribonucleoprotein complexes”, and “ribosomal subunits” (*p* < 0.05) (Figure 3A). When examining the SLA versus LLA group, DEGs were significantly enriched in Gene Ontology (GO) terms associated with cellular localization, including “cytoplasmic part”, ”cytoplasm”, and “extracellular region” (*p* < 0.05) (Figure 3B). In the comparison of SLA and MLA, DEGs were significantly enriched in the GO term for “electron transfer activity” within the molecular function category (*p* < 0.05) (Figure 3C).

### 3.4. KEGG Pathway Enrichment of Differentially Expressed Genes

**Figure 4:**
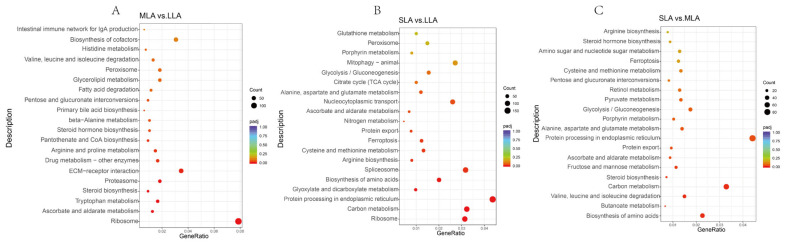
Bubble charts of KEGG pathway enrichment analysis for DEGs. (**A**): KEGG enrichment bubble chart for DEGs in MLA vs. LLA; (**B**): KEGG enrichment bubble chart for DEGs in SLA vs. LLA; (**C**): KEGG enrichment bubble chart for DEGs in SLA vs. MLA. The bubble size corresponds to the number of genes, and the color represents the enrichment significance. The detailed lists of KEGG pathway annotation of differentially expressed genes are provided in Supplementary Table S6.

In the analysis comparing MLA and LLA, differentially expressed genes (DEGs) showed significant enrichment in various pathways, such as “ribosome”, “tryptophan metabolism”, “steroid biosynthesis”, “proteasome”, and “extracellular matrix (ECM)–receptor interaction” (*p* < 0.05) (Figure 4A, Table S4). When comparing SLA to LLA, DEGs were notably enriched in pathways associated with metabolism and cellular functions, including “ribosome”, “carbon metabolism”, “protein processing in the endoplasmic reticulum”, “glyoxylate and dicarboxylate metabolism”, and “biosynthesis of amino acids” (*p* < 0.05) (Figure 4B, Table S5). In the SLA vs. MLA comparison, DEGs were significantly enriched in pathways such as “biosynthesis of amino acids”, “butanoate metabolism”, “valine, leucine, and isoleucine degradation”, “carbon metabolism”, and “steroid biosynthesis” (*p* < 0.05) (Figure 4C).

### 3.5. RT-qPCR Validation of Selected Differentially Expressed Genes

**Figure 5:**
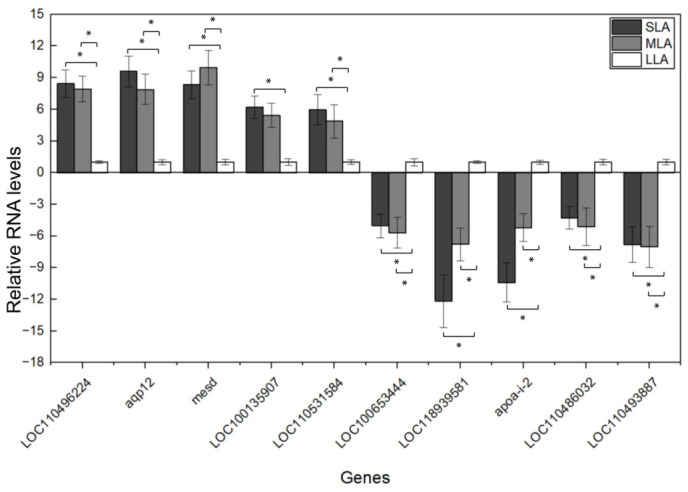
RT-qPCR validation of selected DEGs. Relative RNA levels (mean ± SD, *n* = 3 per group). The RNA levels are expressed relative to the geometric mean of the reference gene *β-actin*, with the LLA group serving as the calibrator (set to 1.0) for each gene examined. The parts with statistically significant differences between groups *(p* < 0.05) are marked with asterisks (*). The asterisks are positioned lower relative to the bar chart, indicating SLA vs. MLA; and higher relative to the bar chart, indicating MLA vs. LLA. All pairwise comparisons were conducted using a two-tailed Student’s *t*-test.

To assess the reliability of the RNA-seq data, we performed RT-qPCR on a subset of ten DEGs that exhibited substantial fold changes among the three body-length groups. The RT-qPCR results showed expression trends consistent with the RNA-seq FPKM values across all three groups (SLA, MLA, and LLA) (Figure 5).

Specifically, *LOC110496224*, *aqp12*, *mesd*, *LOC100135907*, and *LOC110531584* exhibited significantly higher relative RNA levels in the SLA and MLA groups than in the LLA group, displaying a consistent downward trend from small to large body length. In contrast, *LOC100653444*, *LOC118939581*, *apoa-i2*, *LOC110486032*, and *LOC110493887* showed the opposite pattern, with significantly lower expression (all negative values) in the SLA and MLA groups relative to LLA, indicating relative upregulation of these genes in the LLA group. Notably, *LOC118939581* and *apoa-i2* exhibited the most pronounced downregulation in the SLA group, suggesting that these genes may play a more prominent regulatory role in physiological processes associated with smaller body length.

Collectively, these RT-qPCR validation results closely matched the expression patterns observed in the transcriptomic data, confirming the reliability of the RNA-seq dataset and supporting the downstream conclusions drawn from the transcriptomic analyses.

### 3.6. Data Characteristics Metabolomics

**Figure 6:**
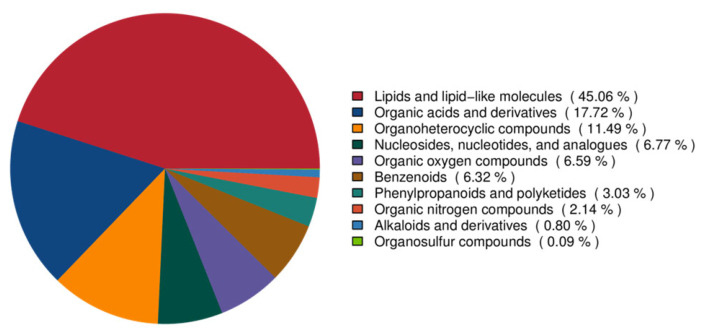
Pie chart of the compositional classification of metabolites identified in liver samples. A total of 1123 distinct metabolites were detected and categorized into ten main classes. The five most abundant categories were lipids and lipid-like molecules (45.06%), organic acids and derivatives (17.72%), organoheterocyclic compounds (11.49%), nucleosides, nucleotides, and analogs (6.77%), and organic oxygen compounds (6.59%). The detailed lists of the identified metabolites and their corresponding classification are provided in Supplementary Table S7.

In the analysis of liver samples, we identified 1123 distinct metabolites, which were organized into ten different categories (Figure 6, Table S7). The five most prevalent categories, ranked by their abundance, included lipids and lipid-like substances (45.06%), organic acids and their derivatives (17.72%), organic heterocyclic substances (11.49%), nucleotides, nucleosides, and their analogs (6.77%), and organic compounds containing oxygen (6.59%).

### 3.7. Principal Component Analysis and Orthogonal Partial Least Squares-Discriminant Analysis of Metabolomics Data

**Figure 7:**
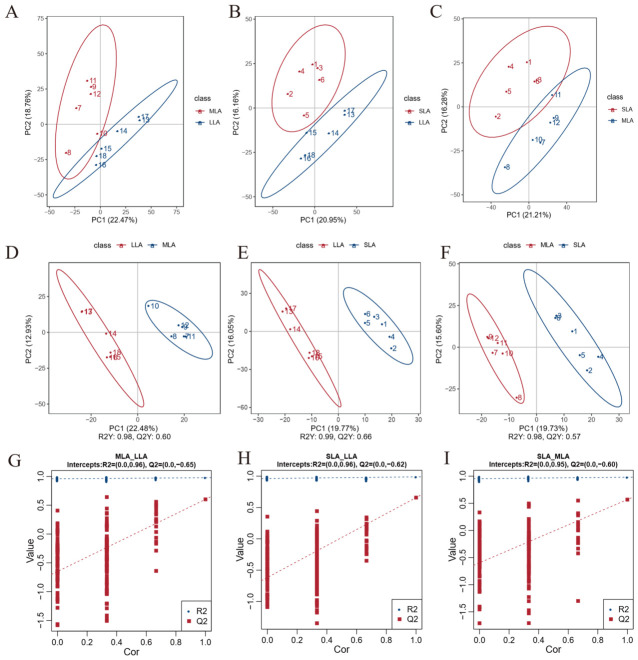
Multivariate statistical analysis of metabolomics data from triploid rainbow trout liver tissues across three body size comparisons. (**A**–**C**) PCA score plots for pairwise comparisons: (**A**): MLA vs. LLA; (**B**) SLA vs. LLA; (**C**): SLA vs. MLA. (**D**–**F**): OPLS-DA score plots for the corresponding pairwise comparisons. (**D**): MLA vs. LLA; (**E**): SLA vs. LLA; (**F**): SLA vs. MLA (**G**–**I**): Permutation test plots (200 iterations) for OPLS-DA model validation. The X-axis (Cor) represents the correlation coefficient between the original and permuted response vectors, representing the similarity between permuted and true class labels. The Y-axis shows R^2^ (goodness of fit, blue dots) and Q^2^ (predictive ability, red dots) values of each permuted model. Dashed lines represent regression fits to Q^2^ values. The original model R^2^ and Q^2^ values are marked with distinct symbols. Q^2^ intercepts below zero indicate that the models are not overfitted and that the observed group separation is statistically reliable. (**G**): Permutation test plot for MLA vs. LLA; (**H**): permutation test plot for SLA vs. LLA; (**I**): permutation test plot for SLA vs. MLA.

Principal component analysis (PCA) demonstrated distinct separations between the MLA and LLA, SLA and LLA, as well as SLA and MLA groups, with samples clustering tightly within each category (Figure 7A–C). This observation suggests a high level of consistency within the groups and unique metabolite profiles across varying size classifications. Additionally, orthogonal partial least squares–discriminant analysis (OPLS-DA) reinforced the presence of unique metabolic profiles among the three groups. The OPLS-DA score plots illustrated clear distinctions in the MLA vs. LLA, SLA vs. LLA, and SLA vs. MLA comparisons, highlighting significant variations in liver metabolism related to body size (Figure 7D–F). The OPLS-DA model’s reliability was established through permutation tests (200 iterations), with R^2^ and Q^2^ values nearing 1, indicating strong model performance, stability, and predictive accuracy, without signs of overfitting. This validated the model’s effectiveness in examining metabolic variations among different sizes of rainbow trout (Figure 7G–I). 

### 3.8. Analysis of Differentially Abundant Metabolites

**Figure 8:**
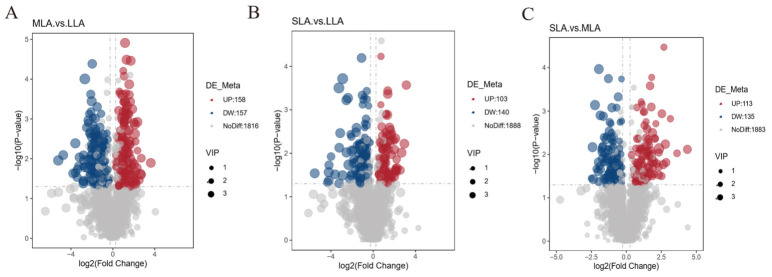
Volcano plots of DAMs. (**A**): Volcano plot for MLA vs. LLA; (**B**): volcano plot for SLA vs. LLA; (**C**): volcano plot for SLA vs. MLA. Red points indicate up-regulated metabolites, and blue points indicate down-regulated metabolites. The detailed lists of differentially accumulated metabolites (DAMs) are provided in Supplementary Table S8.

An analysis of metabolites comparing the MLA vs. LLA groups identified 315 differentially abundant metabolites (DAMs), including 158 DAMs up-regulated and 157 down-regulated DAMs (Figure 8A, Table S8). In a similar vein, the examination of SLA vs. LLA groups revealed 243 DAMs, including 103 metabolites up-regulated metabolites and 140 down-regulated metabolites (Figure 8B, Table S8). Additionally, the SLA vs. MLA comparison demonstrated the most substantial metabolic alterations, yielding 248 DAMs, among which 113 metabolites were up-regulated while 135 were down-regulated (Figure 8C, Table S8).

### 3.9. Functional Annotation of Differentially Abundant Metabolites

**Figure 9:**
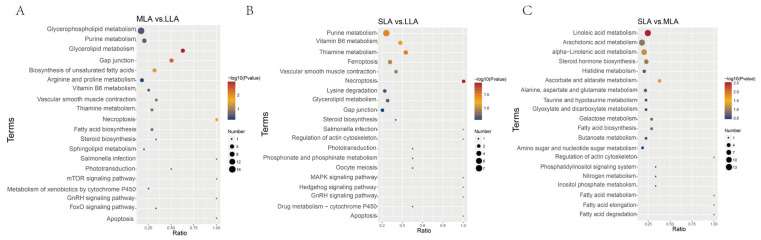
Bubble charts of KEGG pathway enrichment analysis for DAMs. (**A**): KEGG enrichment bubble chart for DAMs in MLA vs. LLA; (**B**): KEGG enrichment bubble chart for DAMs in SLA vs. LLA; (**C**): KEGG enrichment bubble chart for DAMs in SLA vs. MLA. The detailed lists of KEGG pathway enrichment results for DAMs are provided in Supplementary Table S9.

In the comparison between MLA and LLA, DAMs showed a notable enrichment in the pathways related to “glycerolipid metabolism,” “gap junction,” “biosynthesis of unsaturated fatty acids,” and “necroptosis” (*p* < 0.05) (Figure 9A, Table S9).

For the SLA vs. LLA comparison, DAMs showed a notable enrichment in pathways such as “necroptosis,” “thiamine metabolism,” “purine metabolism,” and “vitamin B6 metabolism” (*p* < 0.05) (Figure 9B, Table S9).

Similarly, in the SLA vs. MLA comparison, it was found that DAMs showed a notable enrichment in the pathways related to “linoleic acid metabolism” and “ascorbate and aldarate metabolism” pathways (*p* < 0.05) (Figure 9C, Table S9).

### 3.10. Integrated Analysis of Multi-Omics Data

**Figure 10:**
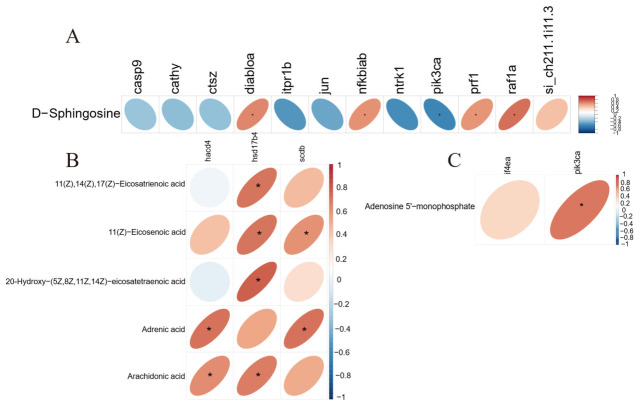
Heatmaps of correlation analysis between DEGs and DAMs in the MLA vs. LLA group. The X-axis represents genes, and the Y-axis represents metabolites. The asterisks (*) indicate statistically significant correlations (*p* < 0.05). (**A**): apoptosis pathway; (**B**): pathway of biosynthesis of unsaturated fatty acids; (**C**): mTOR signaling pathway; information on genes and metabolites involved in each pathway in the MLA vs. LLA group is provided in Supplementary Table S11.

**Figure 11:**
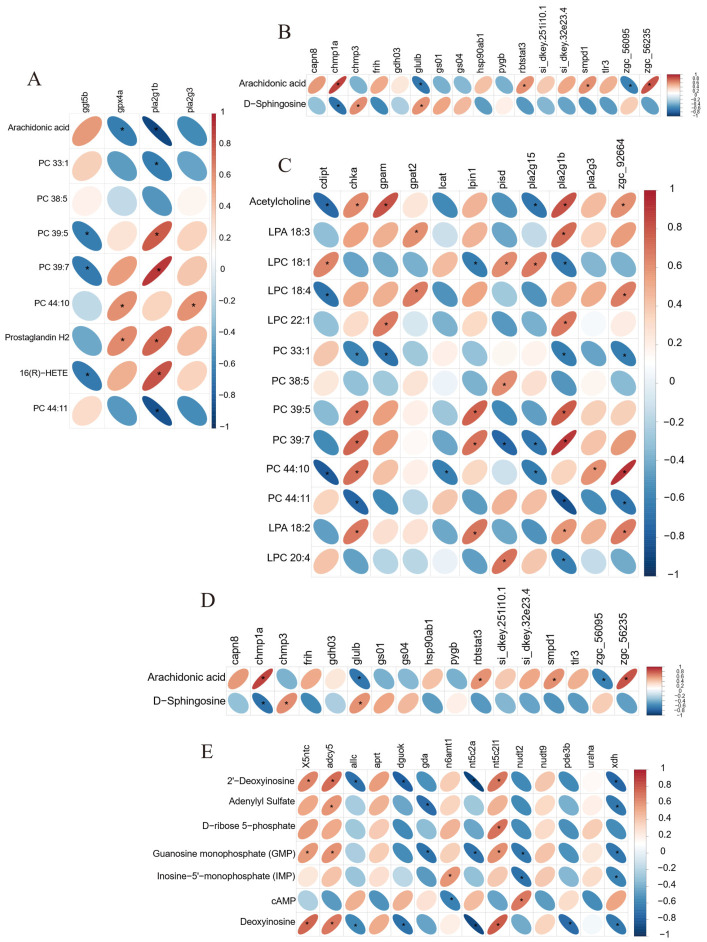
Heatmaps of correlation analysis between DEGs and DAMs in each pathway in the SLA vs. LLA group. The asterisks (*) indicate statistically significant correlations (*p* < 0.05) (**A**): Arachidonic acid metabolism pathway; (**B**): ferroptosis pathway; (**C**): glycerophospholipid metabolism pathway; (**D**): necroptosis pathway; (**E**): purine metabolism pathway. Information on genes and metabolites involved in each pathway in the SLA vs. LLA is provided in Supplementary Table S12.

**Figure 12:**
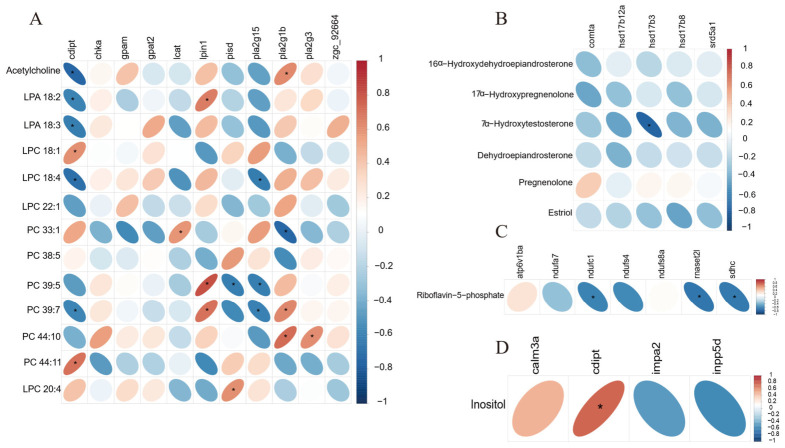
Heatmaps of correlation analysis between DEGs and DAMs in each pathway in the SLA vs. MLA group. The asterisks (*) indicate statistically significant correlations (*p* < 0.05) (**A**): Glycerophospholipid metabolism pathway; (**B**): steroid hormone biosynthesis pathway; (**C**): oxidative phosphorylation pathway; (**D**): phosphatidylinositol signaling system pathway; information on genes and metabolites involved in each pathway in the SLA vs. MLA is provided in Supplementary Table S13.

To determine whether the transcriptomic and metabolomic changes were coordinately regulated at the pathway level, we performed integrated gene–metabolite correlation analysis using DEGs and DAMs co-enriched within the same KEGG pathway for each pairwise comparison (Table S10). For each shared pathway, Pearson correlation coefficients were calculated between the expression levels of the annotated DEGs and the relative abundances of the annotated DAMs. The resulting gene–metabolite correlation networks are presented as heatmaps (Figure 10, Figure 11 and Figure 12), split into three subsections according to the three pairwise comparisons. All correlation data are provided in Supplementary Tables S11–S13.

#### 3.10.1. MLA vs. LLA: Apoptosis, Fatty Acid Remodeling, and Nutrient Sensing

In the MLA vs. LLA comparison, integrated analysis focused on three co-enriched pathways: apoptosis, biosynthesis of unsaturated fatty acids, and mTOR signaling pathway. Within the apoptosis pathway, D-sphingosine—a key sphingolipid metabolite involved in cell death signaling—showed positive correlations with pro-apoptotic regulators (*diabloa*, *nfkbiab*, *prf1*, *raf1a*) and a negative correlation with *pik3ca*, a central component of the PI3K-AKT survival axis (Figure 10A). This pattern suggests that sphingolipid-mediated apoptotic signaling may be more active in medium-sized fish compared to larger individuals.

In the pathway of the biosynthesis of unsaturated fatty acids, several positive correlations were identified between genes involved in fatty acid elongation/desaturation and their corresponding PUFA products (Figure 10B). Specifically, *hacd4* correlated with adrenic acid and arachidonic acid; *hsd17b4* showed correlations with multiple eicosanoid precursors; and scdb correlated with 11(Z)-eicosenoic acid and adrenic acid. In the mTOR signaling pathway, *pik3ca* positively correlated with adenosine 5′-monophosphate(AMP) (Figure 10C), suggesting a link between nutrient sensing and energy status in medium-sized fish.

#### 3.10.2. SLA vs. LLA: Oxidative Stress, Membrane Remodeling, and Cell Death

The SLA vs. LLA comparison yielded the most extensive integrated networks, spanning five co-enriched pathways: arachidonic acid metabolism, ferroptosis, glycerophospholipid metabolism, necroptosis, and purine metabolism (Table S12). These pathways collectively capture alterations in eicosanoid signaling, oxidative lipid-induced cell death, membrane phospholipid remodeling, inflammatory cell death, and nucleotide turnover.

In the arachidonic acid metabolism pathway, a central regulatory hub emerged involving *pla2g1b*, *gpx4a*, and their associated lipid mediators (Figure 11A). *pla2g1b* showed positive correlations with prostaglandin H2 and 16(R)-HETE, while *gpx4a* correlated positively with prostaglandin H2 but negatively with arachidonic acid itself—consistent with a regulatory axis where *pla2g1b* liberates arachidonic acid from membrane phospholipids driving eicosanoid production, while *gpx4a* functions as an antioxidant counterbalance.

Within the ferroptosis pathway (Figure 11B), arachidonic acid showed negative correlations with ferroptosis suppressor genes (*acsl4a*, *gpx4a*) and positive correlations with pro-ferroptotic markers (*hmox1a*, *ncoa4*, *tfr1a*), indicating that smaller fish may be more susceptible to oxidative lipid-mediated cell death.

The glycerophospholipid metabolism pathway exhibited the most complex network (Figure 11C), with key enzymes including *chka*, *lpin1*, and multiple phospholipase A2 members (*pla2g1b*, *pla2g3*) showing extensive correlations with various phosphatidylcholines (PCs) and lysophosphatidylcholines (LPCs). This suggests that membrane phospholipid remodeling is substantially more active in smaller fish compared to larger ones.

In the necroptosis pathway (Figure 11D), arachidonic acid correlated positively with several necroptotic regulators (*chmp1a*, *rbtstat3*, *smpd1*), while D-sphingosine showed correlations with *chmp3* and *glulb*, implicating both lipid mediators in inflammatory cell death signaling.

Finally, in purine metabolism (Figure 11E), multiple correlations were identified between purine metabolic enzymes and nucleotide metabolites (GMP, IMP, cAMP, deoxyinosine), reflecting disruptions in nucleotide pools and ATP turnover consistent with elevated energy demands and oxidative stress in smaller fish.

#### 3.10.3. SLA vs. MLA: Steroid Metabolism, Membrane Remodeling, and Energy Production

In the SLA vs. MLA comparison, four co-enriched pathways were analyzed: steroid hormone biosynthesis, glycerophospholipid metabolism, oxidative phosphorylation, and phosphatidylinositol signaling system (Table S13).

For glycerophospholipid metabolism (Figure 12A), *cdipt*, *lcat*, *lpin1*, *pisd*, *pla2g15*, and multiple PLA2 members showed distinct correlation patterns with PC and LPC species, indicating that membrane remodeling patterns differ between small and medium-sized fish.

In the steroid hormone biosynthesis pathway (Figure 12B), *hsd17b3* exhibited a significant negative correlation with 7α-hydroxytestosterone, suggesting suppressed androgenic steroid production in smaller fish relative to medium-sized individuals—a pattern consistent with size-associated steroid-mediated energy repartitioning.

In oxidative phosphorylation (Figure 12C), *ndufc1*, *rnaset2l*, and *sdhc* were all negatively correlated with riboflavin-5′-phosphate, while in the phosphatidylinositol signaling system (Figure 12D), CDIPT showed a positive correlation with inositol. These findings collectively point to differences in mitochondrial energy metabolism and phosphoinositide-mediated signaling between small and medium fish.

## 4. Discussion

While body size serves as a convenient and practical metric for stratifying individuals in aquaculture settings, we acknowledge that it is an integrative proxy rather than a direct causal factor. In this study, all experimental fish originated from a single full-sibling family, were of the same year class (and thus comparable age), and were reared under identical conditions from fertilization through sampling. The use of triploid fish further eliminates sex-related maturation effects, as triploid sterility ensures that reproductive development does not divert energy from somatic growth or alter physiological baselines [49]. Rearing history was standardized across all individuals, with identical stocking density, feeding regimes, and thermal history prior to field deployment. Under these controlled conditions, size variation within the sampled population primarily reflects intrinsic genetic differences in growth rate rather than disparities in age, sex, or rearing background [50]. Therefore, in the context of our experimental design, body size serves as a legitimate and informative stratification variable for investigating thermal adaptation. Nonetheless, we recognize that body size per se may be a proxy for underlying physiological states—such as metabolic rate, energy reserves, or oxidative stress load—that more directly determine adaptive capacity. Future studies incorporating longitudinal tracking of individual fish across seasonal thermal cycles would help clarify whether the unique regulatory programs in large individuals reflect irreversible developmental programming or plastic responses to size-specific heat exposure. Additionally, incorporating condition indices such as Fulton’s condition factor (K) or hepatosomatic index (HSI) in future work could further disentangle the contributions of nutritional status and energy allocation from the effects of body size [51].

### 4.1. Size-Associated Transcriptomic Changes

The distinct group-specific differentially expressed genes (DEGs)—975 for SLA, 571 for MLA, and 863 for LLA—indicate a complex, size-related trend in transcriptional changes. The smallest fish (SLA) exhibited the most significant alterations in gene expression, likely due to their greater surface-area-to-volume ratio, which enhances heat absorption and results in increased thermal stress relative to body mass [52]. This size-associated thermal sensitivity is well documented in salmonids: smaller individuals have higher mass-specific metabolic rates and lower thermal buffering capacity than larger conspecifics [53]. The notably higher count of LLA-specific DEGs (863) in comparison to MLA (571) may suggest size-threshold phenomena, where the largest fish activate unique regulatory mechanisms that do not follow a linear progression from the intermediate sizes, highlighting a need for further exploration [54].

Analysis of KEGG pathway enrichment highlighted significant variations in metabolic focus among different size categories. Notably, differentially expressed genes assigned to the “ribosome” pathway were significantly enriched in both the MLA vs. LLA and SLA vs. LLA comparisons, but not in the SLA vs. MLA comparison [55,56]. This shared enrichment of ribosome-related genes in comparisons involving LLA suggests that maintaining protein balance represents a common adaptive challenge for both small and medium fish relative to the largest size class under summer heat stress.

Energy metabolism pathways exhibited a clear association on size. The pathways related to “carbon metabolism” and “biosynthesis of amino acids” were notably enriched in comparisons involving SLA (SLA vs. LLA and SLA vs. MLA), but not in MLA vs. LLA. This pattern suggests that smaller fish may increase catabolic and biosynthetic activity under heat stress. One possible explanation is that reduced feed intake coupled with elevated energy expenditure creates a metabolic deficit that smaller fish—having limited energy reserves—attempt to compensate for through enhanced endogenous catabolism [57,58]. The additional enrichment of “tryptophan metabolism” (MLA vs. LLA) and “valine, leucine and isoleucine degradation” (SLA vs. MLA) points to branched-chain amino acids breakdown as a potential supplementary energy source, particularly in smaller fish [59,60]. Larger fish (LLA) did not show comparable enrichment in these pathways, which may reflect a more stable metabolic state associated with greater energy reserves and lower mass-specific metabolic rates. The enrichment of “steroid biosynthesis” in both MLA vs. LLA and SLA vs. MLA comparisons points to a size-associated hormone influences on energy distribution—a pattern that may be particularly relevant in triploid fish, which divert energy from reproductive development to somatic growth [61].

The heat- and immune-related gene expression patterns were not the primary focus of our experimental design. Given that our transcriptomic screening was designed to capture global transcriptional changes rather than to specifically interrogate immune gene panels, a detailed characterization of immune-related genes was not pursued in our results. Future studies employing targeted immune gene panels or challenge experiments would be valuable to further dissect the size-associated aspects of immunocompetence under thermal stress.

### 4.2. Size-Associated Metabolomic Profiles

Metabolomic analysis indicated that lipids and similar compounds were the predominant class, accounting for 45.06% of all identified metabolites, underscoring the crucial role of lipid metabolism in the thermal stress response of rainbow trout [62]. The “biosynthesis of unsaturated fatty acids” pathway emerged as a noteworthy candidate, as membrane fluidity—modulated by fatty acid unsaturation—represents a well-established mechanism for thermal acclimation [63,64]. Interestingly, this pathway was enriched only in the MLA vs. LLA comparison, but not in those involving SLA. This observation raises the possibility that medium-sized fish preferentially employ targeted membrane remodeling as a primary adaptive strategy, whereas smaller fish may engage additional or alternative mechanisms. In contrast, SLA vs. LLA was uniquely enriched in “necroptosis”, “purine metabolism”, and “thiamine metabolism”. The enrichment of necroptosis-related pathways is consistent with the view that smaller fish may experience more severe cellular damage under identical thermal conditions, potentially due to their higher heat load per unit mass. The altered purine metabolites observed in SLA fish (e.g., GMP, deoxyinosine) may reflect disruptions in nucleotide pools and ATP turnover, which are often associated with elevated oxidative stress and energy depletion [65,66]. The SLA vs. MLA comparison showed unique enrichment of “linoleic acid metabolism” and “ascorbate and aldarate metabolism”, suggesting that PUFA-derived bioactive lipid signaling and antioxidant metabolism differ between small and medium fish [67,68].

### 4.3. Integrated Gene–Metabolite Regulatory Networks

A comprehensive multi-omics approach was employed to develop gene–metabolite relationships within pathways enriched at both the transcriptomic and metabolomic levels. The metabolism of arachidonic acid (AA) was selected for closer examination because of its position at the intersection of ferroptosis and necroptosis pathways, both of which were highlighted in the metabolomic analysis of SLA and LLA [69]. The AA pathway as also plays a crucial role in regulating inflammation and oxidative cell fate decision. In the SLA vs. LLA comparison, *pla2g1b* (phospholipase A2) showed positive correlations with both prostaglandin H2 and 16(R)-HETE, while gpx4a (glutathione peroxidase 4) correlated positively with prostaglandin H2 but negatively with AA itself. This correlation pattern is consistent with a regulatory axis in which *pla2g1b* may liberate AA from membrane phospholipids, promoting eicosanoid production, while *gpx4a* could function as an antioxidant counterbalance [70,71]. These correlations also extended to ferroptosis-related genes: *acsl4a*—which facilitates PUFA incorporation into membrane phospholipids and is associated with ferroptosis susceptibility—correlated positively with AA, as did hmox1a, a marker of oxidative stress and iron metabolism [72,73,74]. Importantly, this AA–ferroptosis correlation network was most prominent in SLA vs. LLA and largely absent in MLA vs. LLA, suggesting that smaller fish may experience greater oxidative and cell-death-related pressure—a potentially size-associated distinction [75,76,77]. We emphasize, however, that these correlations do not establish causality; they identify candidate regulatory relationships that warrant functional validation in future studies.

The “glycerophospholipid metabolism” pathway exhibited the most extensive gene–metabolite correlation network and was enriched in SLA vs. LLA and SLA vs. MLA, but not in MLA vs. LLA. Glycerophospholipids are fundamental structural components of cellular membranes, and their remodeling can influence membrane physical properties and signaling functions under thermal stress [78,79]. In SLA vs. LLA, key enzymes including *chka* (choline kinase alpha), *lpin1*, and multiple phospholipase A2 members (*pla2g1b*, *pla2g3*) showed complex correlations with a broad range of phosphatidylcholines (PCs) and lysophosphatidylcholines (LPCs). These correlation patterns are suggestive of extensive phospholipid remodeling [80]. In SLA vs. MLA, a similar but quantitatively distinct pattern emerged, with *chka* and *lpin1* again showing central roles but correlating with partially different PC/LPC species. This observation raises the possibility that while membrane remodeling is common to smaller fish, the specific compositional targets may differ between SLA and MLA [81]. Large fish (LLA) exhibited fewer and simpler gene–metabolite correlations within this pathway, which may indicate a more stable glycerophospholipid profile under thermal stress. Viewed together, these patterns are consistent with a size-associated gradient in membrane lipid remodeling, with the most extensive changes observed in smaller fish [82].

The “steroid biosynthesis” pathway showed significant enrichment at both omics levels, particularly in the SLA vs. MLA. The gene *hsd17b3* (17beta-hydroxysteroid dehydrogenase type 3) exhibited a significant negative correlation with its product 7alpha-hydroxytestosterone. This correlation could reflect suppressed androgenic steroid production in smaller fish relative to medium-sized ones under thermal stress [83]. We examined this pathway because triploid rainbow trout are reproductively sterile and do not invest energy in gonadogenesis; hormonal modulation in these fish is therefore more plausibly linked to somatic energy repartitioning and environmental acclimation than to reproductive regulation [84]. The size-specific nature of this correlation—absent in both SLA vs. LLA and MLA vs. LLA—suggests that small and medium fish may exhibit distinct steroid-influenced metabolic states. This could represent a hormonal mechanism for balancing energy distribution between maintenance and thermal adaptation within certain weight categories [85,86].

The integration of multi-omics data reveals size-associated patterns in thermal adaptation. Smaller fish (SLA) display the most extensive and resource-intensive responses, characterized by broad transcriptional changes, enhanced energy catabolism, substantial membrane lipid alterations, and correlations with cell death and inflammatory pathways. This comprehensive response is likely related to their higher mass-specific heat load and limited energy reserves. Medium-sized fish (MLA) appear to adopt a more balanced approach, with enrichment of protein homeostasis pathways (ribosome) and selective membrane unsaturation, alongside fewer associations with cell-death pathways. Larger fish (LLA), by contrast, show a more restrained molecular profile, which may reflect greater thermal buffering capacity or a more effective stress-response system. These size-associated patterns identify candidate molecular targets for each size category that could inform tailored management and selective breeding strategies in rainbow trout farming. The interpretations in this study are based on correlational data and require functional validation through targeted perturbation experiments. Furthermore, while our study design—using fish of the same age, genetic background, and rearing history—allows us to attribute observed differences to size rather than to confounding factors, we acknowledge that the correlational nature of multi-omics data does not permit definitive conclusions about causality. Whether these patterns represent adaptive responses to heat stress or intrinsic metabolic differences that happen to correlate with size remains an open question that will require longitudinal and functional studies to resolve.

While the present study was designed to investigate size-associated thermal adaptation mechanisms specifically within triploid rainbow trout, without including diploid comparisons. Previous studies have documented differential responses between diploid and triploid rainbow trout under thermal stress. At chronic high temperature (21 °C), triploids showed significantly higher mortality (68.5%) than diploids (39%) over three weeks [87]. Under acute thermal stress, diploids and triploids exhibited differential antioxidant enzyme induction patterns, with triploids mounting stronger responses at 25 °C [88]. Triploids also show greater hematological susceptibility to thermal stress, with more pronounced changes in white blood cell counts and hemoglobin levels following temperature elevation [89]. These observations highlight that ploidy itself is an important factor modulating thermal physiology. Future investigations incorporating direct comparisons between diploid and triploid rainbow trout under identical thermal stress conditions should be conducted and would help determine whether the size-associated molecular patterns observed in the present study are triploid-specific phenomena or reflect general principles of thermal adaptation that apply across ploidy levels.

A key consideration in interpreting our findings is that we sampled fish only under heat stress conditions, without a corresponding control group at optimal temperature. This means we cannot definitively attribute the observed molecular differences to high-temperature adaptation per se, as opposed to intrinsic size-related differences in baseline gene expression and metabolite profiles. We acknowledge that our cross-sectional design cannot formally distinguish heat-adaptive changes from size-correlated variation. Future studies incorporating a control group at optimal temperature (e.g., 16–18 °C) across the same size classes would be valuable to parse the relative contributions of thermal stress versus intrinsic size effects. Such paired designs would allow direct comparison of the same size groups under control and heat-stressed conditions, thereby clarifying which molecular signatures are truly attributable to high-temperature adaptation.

## 5. Conclusions

This study provides a comprehensive multi-omics characterization of the molecular mechanisms underlying high-temperature adaptation in triploid rainbow trout, revealing size-specific transcriptional and metabolic pathways. The integrated analysis demonstrates that thermal adaptation involves coordinated regulation of proteostasis maintenance (ribosome pathway, protein processing in the ER), membrane lipid re-modeling (glycolipid metabolism, biosynthesis of unsaturated fatty acids, glycerophospholipid metabolism), inflammatory and cell death signaling (apoptosis, ferroptosis, necroptosis), and energy metabolism (oxidative phosphorylation, carbon metabolism). The extensive correlation networks between differentially expressed genes and metabolites provide molecular evidence for the interplay between these pathways in mediating thermal tolerance. Importantly, smaller fish (SLA) exhibit more extensive transcriptomic changes, greater reliance on eicosanoid signaling and ferroptosis pathways, and potential mitochondrial dysfunction compared to larger individuals. These findings not only enhance our understanding of the adaptive strategies of cold-water fish in the context of global warming but also provide potential molecular markers for selective breeding and aquaculture management. The size-associated heterogeneity in stress responses highlights the need for size-specific temperature management strategies to optimize the health and productivity of triploid rainbow trout in aquaculture settings. Our multi-omics screening did not specifically target immune-related genes. From a management perspective, this underscores the importance of mitigating thermal stress—particularly for smaller, more-vulnerable-size classes—to minimize immune–metabolic trade-offs that may increase susceptibility to opportunistic pathogens in densely stocked systems.

## Figures and Tables

**Table 1:** Primer sequences of genes used in RT-qPCR.

Genes	Description	Primer Sequence (5′→3′)
*LOC110496224*	H-2 class I histocompatibility antigen, Q9 alpha chain	Forward primer: ATGTACGGTTGTGAGTGGGAT
		Reverse primer: GTATGTGGACTATGGGAAGAGCA
*aqp12*		Forward primer: ACTTTCTCGCTGTTGTGGTG
		Reverse primer: GTCAGGGAAGGGTTTCCGGT
*mesd*		Forward primer: ATGTCCCATGttgtccctcc
		Reverse primer: TGTCATCTTTCTTGGCGGCC
*LOC100135907*	vitelline envelope protein gamma	Forward primer: ATGGCGATGAAGTGGAGTGT
		Reverse primer: GGTTCTCGCCTCTGAGCT
*LOC110531584*	purine nucleoside phosphorylase 4b (pnp4b in the KEGG database)	Forward primer: ATGAGGACTGCAAGGGCAC
		Reverse primer: ATCCCTCGTAGAGGTGGAAG
*LOC100653444*	hepcidin-like antimicrobial peptide	Forward primer: ATGAAGGCCTTCAGTGTTGC
		Reverse primer: TCAGAATTTGCAGCAGAAGCC
*LOC118939581*	complement C3-like protein	Forward primer: ATTAGGGGTGCAGACCAAGG
		Reverse primer: AGACACAGCAAGTTGCCG
*apoa-i-2*		Forward primer: ATGCAATTCCTGGCTCTTGCA
		Reverse primer: CGGTCTGGGCATACTGC
*LOC110486032*	vitamin D(3) 25-hydroxylase	Forward primer: ATCCTTGGCAACCTGCTACA
		Reverse primer: ATAATTGGCCATGACGATCCC
*LOC110493887*	1,25-dihydroxyvitamin D(3) 24-hydroxylase, mitochondrial	Forward primer: ACATCTTCAGTGTGCGTCC
		Reverse primer: CGATTCAAAGGAGCCGAGT
*β-actin*		Forward primer: TACAACGAGCTGAGGGTGGC
		Reverse primer: GGCAGGGGTGTTGAAGGTCT

**Table 2:** Statistical summary of sequencing data.

Sample	Raw_Reads	Raw_Bases	Clean_Bases	Q20	Q30	GC_*P*ct
SLA1	49,771,516	7.47 G	7.08 G	98.67	96.64	47.3
SLA2	47,789,096	7.17 G	6.72 G	98.73	96.79	46.36
SLA3	46,039,826	6.91 G	6.46 G	98.56	96.46	46.19
SLA4	41,136,196	6.17 G	5.88 G	97.75	94.01	46.35
SLA5	44,701,270	6.71 G	6.27 G	98.62	96.54	46.15
SLA6	43,440,740	6.52 G	6.06 G	98.61	96.54	46.3
MLA1	40,779,796	6.12 G	5.85 G	97.7	93.67	47.68
MLA2	46,280,704	6.94 G	6.57 G	98.76	96.82	48.03
MLA3	43,207,254	6.48 G	6.01 G	98.5	96.08	46.97
MLA4	409,462,80	6.14 G	5.64 G	98.68	96.69	46.76
MLA5	497,086,08	7.46 G	7.43 G	98.8	96.92	48.24
MLA6	483,219,38	7.25 G	7.08 G	98.81	96.71	45.96
LLA1	41,190,394	6.18 G	5.86 G	98.75	96.81	47.69
LLA2	40,917,924	6.14 G	5.86 G	98.75	96.8	47.6
LLA3	47,857,156	7.18 G	6.88 G	98.72	96.73	48.11
LLA4	39,310,064	5.9 G	5.62 G	98.73	96.77	46.38
LLA5	40,554,994	6.08 G	5.69 G	98.86	96.99	48.54
LLA6	46,145,486	6.92 G	6.64 G	97.91	94.11	47.78

## Data Availability

The raw data generated with transcriptome sequencing in this study are available in the NCBI SRA database under BioProject Accession Number PRJNA1367965. Available online: https://dataview.ncbi.nlm.nih.gov/object/PRJNA1367965 accessed on 1 January 2026). The LC-MS/MS data have been deposited via MetaboLights (https://www.ebi.ac.uk/metabolights/MTBLS14507 accessed on 18 May 2026).

## Supplementary Materials

The following supporting information can be downloaded at https://www.mdpi.com/article/10.3390/biology15141194/s1, Table S1: Gene IDs and annotations of expressed genes; Table S2: The detailed lists of differentially expressed genes; Table S3: The detailed lists of GO enrichment of differentially expressed genes (DEGs); Table S4: The detailed lists of KEGG pathway annotation for differentially expressed genes in the MLA vs. LLA; Table S5: The detailed lists of KEGG pathway annotation for differentially expressed genes in the LA vs. LLA; Table S6: The detailed lists of KEGG enrichment of differentially expressed genes(DEGs); Table S7: Summary of all 1123 identified metabolites in liver samples; Table S8: List of differentially accumulated metabolites (DAMs); Table S9: The detailed lists of KEGG enrichment of differentially accumulated metabolites (DAMs); Table S10: The detailed lists of integrated KEGG enrichment of DEGs and DAMs; Table S11: List of genes and metabolites involved in each KEGG pathway in the MLA vs. LLA; Table S12: List of genes and metabolites involved in each KEGG pathway in the SLA vs. LLA; Table S13: List of genes and metabolites involved in each KEGG pathway in the SLA vs. MLA.
